# Supplementary material for: The Role of DNA Methylation in Stroke Recovery
Source: Int J Mol Sci. 2022 Sep 8;23(18):10373. doi: 10.3390/ijms231810373 (PMC9499691; doi:10.3390/ijms231810373)
Supplement: Supplementary file 1 [file ijms-23-10373-s001.zip › ijms-1885986-supplementary.pdf]

Methods

Search strategy of literatures

To identify the relevant publications we conducted systematic searches in the bibliographic databases PubMed and Medline from inception up to April 18, 2022, in collaboration with a medical information specialist. All articles were solicited from the databases using the searching terms “DNA methylation treatment”, “DNA methylation neuroprotection”, “DNA methylation recovery” and “stroke”. A key word search for “DNA methylation treatment/neuroprotection/recovery” were used medical subject headings (MeSH) terms and all fields’ terms. Stroke keyword was searched using MeSH terms. The references of the identified articles were searched for relevant publications. Duplicate articles were excluded. All languages were accepted.

Search number	Query	Search Details	Results
4	#1 OR #2 OR #3	**DNA methylation**: "dna methylation"[MeSH Terms] OR ("dna"[All Fields] AND "methylation"[All Fields]) OR "dna methylation"[All Fields]<br><br>**recovery**: "recoveries"[All Fields] OR "recovery"[All Fields]<br><br>**neuroprotection**: "neuroprotect"[All Fields] OR "neuroprotecting"[All Fields] OR "neuroprotection"[MeSH Terms] OR "neuroprotection"[All Fields] OR "neuroprotective agents"[Pharmacological Action] OR "neuroprotective agents"[MeSH Terms] OR ("neuroprotective"[All Fields] AND "agents"[All Fields]) OR "neuroprotective agents"[All Fields] OR "neuroprotectant"[All Fields] OR "neuroprotectants"[All Fields] OR "neuroprotective"[All Fields] OR "neuroprotectively"[All Fields] OR "neuroprotectives"[All Fields] OR "neuroprotectivity"[All Fields] OR "neuroprotects"[All Fields]<br><br>**treatment**: "therapeutics"[MeSH Terms] OR "therapeutics"[All Fields] OR "treatments"[All Fields] OR "therapy"[Subheading] OR "therapy"[All Fields] OR "treatment"[All Fields] OR "treatment's"[All Fields]<br><br>**stroke**[MeSH Terms]: "stroke"[MeSH Terms]	50
3	(DNA methylation recovery) AND (Stroke[MeSH Terms])	("dna methylation"[MeSH Terms] OR ("dna"[All Fields] AND "methylation"[All Fields]) OR "dna methylation"[All Fields]) AND	13

		("recoveries"[All Fields] OR "recovery"[All Fields]) AND "stroke"[MeSH Terms]	
2	(DNA methylation neuroprotection) AND (stroke[MeSH Terms])	("dna methylation"[MeSH Terms] OR ("dna"[All Fields] AND "methylation"[All Fields]) OR "dna methylation"[All Fields]) AND ("neuroprotect"[All Fields] OR "neuroprotecting"[All Fields] OR "neuroprotection"[MeSH Terms] OR "neuroprotection"[All Fields] OR "neuroprotective agents"[Pharmacological Action] OR "neuroprotective agents"[MeSH Terms] OR ("neuroprotective"[All Fields] AND "agents"[All Fields]) OR "neuroprotective agents"[All Fields] OR "neuroprotectant"[All Fields] OR "neuroprotectants"[All Fields] OR "neuroprotective"[All Fields] OR "neuroprotectively"[All Fields] OR "neuroprotectives"[All Fields] OR "neuroprotectivity"[All Fields] OR "neuroprotects"[All Fields]) AND "stroke"[MeSH Terms]	10
1	(DNA methylation treatment) AND (stroke[MeSH Terms])	("dna methylation"[MeSH Terms] OR ("dna"[All Fields] AND "methylation"[All Fields]) OR "dna methylation"[All Fields]) AND ("therapeutics"[MeSH Terms] OR "therapeutics"[All Fields] OR "treatments"[All Fields] OR "therapy"[MeSH Subheading] OR "therapy"[All Fields] OR "treatment"[All Fields] OR "treatment s"[All Fields]) AND "stroke"[MeSH Terms]	44
